# Post-Traumatic Stress Disorder Symptoms among Journalists Repeatedly Covering COVID-19 News

**DOI:** 10.3390/ijerph18168536

**Published:** 2021-08-12

**Authors:** Gabriella Tyson, Jennifer Wild

**Affiliations:** Oxford Centre for Anxiety Disorders and Trauma, Department of Experimental Psychology, University of Oxford, Oxford OX1 1TW, UK; jennifer.wild@psy.ox.ac.uk

**Keywords:** PTSD, journalists, COVID-19, predictors

## Abstract

The coronavirus pandemic has resulted in many journalists repeatedly covering stories related to human suffering. This study investigates whether these journalists experienced higher rates of psychological distress, post-traumatic stress disorder (PTSD) and depressive symptoms than those who have been working during the pandemic yet covering stories other than COVID-19 and aims to identify what factors may protect journalists from developing trauma-related symptoms. We assessed journalists (*n* = 120) working during the COVID-19 pandemic using self-report measures. Journalists repeatedly covering COVID-19 stories had significantly higher psychological distress (η^2^ = 0.04) and PTSD symptoms (η^2^ = 0.08), but not depression, compared to journalists who did not report on COVID-19. Rumination and numbing in response to unwanted memories predicted PTSD symptoms (R^2^ = 0.53) and may be risk factors for PTSD in this population. Unhelpful resilience appraisals distinguished journalists who reported on COVID-19 and who developed distressing re-experiencing symptoms from those who similarly reported on distressing material and who did not develop symptoms. Targeting resilience appraisals may be helpful in reducing re-experiencing symptoms after trauma exposure.

## 1. Introduction

The role of a journalist is integral to ensuring we as the public are informed about global issues. Journalists frequently work to tight deadlines, investigating stories that cover a wide range of tragedies, which may involve putting themselves at risk [1]. This means they are highly susceptible to stressor-related psychopathology [2,3], and research demonstrates that journalists who repeatedly cover stories of human suffering do indeed suffer higher rates of post-traumatic stress disorder (PTSD) and depression than the general population [2,4]. The COVID-19 pandemic has caused global distress, with journalists reporting on the virus and its consequences since December 2019 [5]. We were interested in documenting the effects of repeated reporting of COVID-19 on the mental health of journalists. We also investigate coping strategies and protective factors related to the severity of symptoms to identify potential targets for preventive interventions.

## 2. Materials and Methods

One hundred and twenty journalists who worked during the COVID-19 pandemic were surveyed. The study was approved by the Medical Sciences Inter-Divisional Research Ethics Committee at the University of Oxford (R68253/RE002). The anonymous survey was created using Qualtrics, a secure survey platform. It was distributed via an anonymous link on social media outlets and via various media organisations’ online newsletters and websites.

Potential participants were given information about the eligibility criteria and aims of the study: namely, to investigate the impact of working in journalism on wellbeing during the COVID-19 pandemic. Participants were able to take part if they could read and respond in English, were over 18 years of age and currently worked in the media sector. 

Demographics. Participants were asked to report their gender, age, marital status, ethnicity, if they had ever received a mental health diagnosis and treatment, whether they worked freelance or for an organisation, their current job title and number of years they had worked in that role. Participants were also asked to indicate whether their role involved repeatedly covering COVID-19 stories.

Trauma Screener. Exposure to traumatic events was measured using a modified version of the Life Events Checklist (LEC) [6]. Participants were asked to indicate whether or not they had experienced, witnessed, or learned about 17 different types of traumatic events. 

PTSD Symptoms. The PTSD Checklist-5 (PCL-5) is a 20-item self-report measure that reflects the DSM-5 symptom criteria for PTSD [7]. A score of 31 or above indicates probable PTSD [8]. The scale evidenced good reliability in this sample (α = 0.95).

Depressive Symptoms. The nine item Patient Health Questionnaire-9 (PHQ-9) was used to assess symptoms of depression [9]. The scale showed good reliability in this sample (α = 0.87).

The Responses to Intrusions Questionnaire (RIQ) was administered to measure coping strategies in response to intrusive memories [10]. It is a 19-item scale comprising of three subscales: suppression, rumination and numbing. Items were scored on a four-point Likert scale from 0 (never) to 3 (always). The scale showed good reliability in this sample (α = 0.89).

Psychological Distress. Psychological distress was measured using the General Health Questionnaire 12-item version (GHQ-12) [11]. This study used the Likert method of scoring, with each item scored from 0 to 3. The scale showed good reliability in this sample (α = 0.88). 

*Resilience.* The Connor-Davidson Resilience Scale (CD-RISC) measured 10 resilience appraisals [12]. Items were measured on a five-point Likert scale from 0 (not true at all) to 4 (true nearly all the time). The scale showed good reliability in this sample (α = 0.90).

### Analysis

The analyses were conducted in SPSS (version 26). At the request of the ethics committee, participants were allowed to skip questionnaires when completing the study. There was some drop off in questionnaire completion as the survey went on, with the PHQ having the largest degree of missingness due to it being at the end of the survey. The missingness for the GHQ (1.7%), trauma exposure (5%), PCL (23.3%), RIQ (24.2%) and PHQ (31.7%) was analysed, and no significant differences were found in the missing cases across the scales, between journalists who had repeatedly covered COVID-19 stories and those who had not. There were no significant differences in those with missing data on any of the demographic measures. Missing data were excluded by listwise deletion. In order to examine group differences in demographic characteristics, PTSD symptoms, depression, psychological distress, and responses to intrusions, one-way ANOVAs and X^2^ analyses (where appropriate) were conducted. Since journalists repeatedly covering COVID-19 experienced greater lifetime trauma exposure than journalists who covered stories other than COVID-19, and there were differences in gender between the two groups, we also conducted ANCOVAs to determine whether or not the findings remained when these differences were controlled for. A multiple linear regression was conducted to examine the responses to intrusions’ subscales as potential predictors of PTSD symptoms. One-way ANOVAs were conducted to examine differences in resilience appraisals between journalists who covered COVID-19 and developed re-experiencing symptoms and those who covered COVID-19 without developing such symptoms. Effect sizes were calculated and reported.

## 3. Results

### 3.1. Participants

One hundred and twenty journalists completed at least 70% of the survey. Demographic information can be seen in Table 1. The sample was comparable in terms of gender and ethnicity to available demographics of UK journalists (*n* = 700) [13]. In regard to gender, participants were invited to select ‘male’ ‘female’ ‘other (please specify if you wish)’ and ‘prefer not to say.’ Since no participants selected the latter two options, male and female genders are reported. A total of 61 participants (50.8%) reported they had repeatedly covered COVID-19 stories and associated consequences over a period of 6 months from May to October 2020, leaving 59 participants (49.2%) who identified as covering news unrelated to COVID-19. There was a significant difference in lifetime trauma exposure between the groups, (*t*(112) = 2.44, *p* = 0.016). There were no significant differences between the groups in terms of having COVID-19 themselves, friends or family having COVID-19, financial difficulties due to COVID-19 or family difficulties due to COVID-19. There were no significant differences between the groups in terms of receiving a mental health diagnosis or having received treatment for a mental health problem. Journalists who repeatedly covered COVID-19 reported significantly longer working hours. They reported seeing death and dying linked to the virus and interviewing people with the COVID-19.

### 3.2. Group Differences

*PTSD Symptoms.* The groups differed significantly with respect to PTSD symptoms, (*F*(1,90) = 7.5, *p* = 0.007). This did not change when gender and trauma exposure were added as covariates (medium effect, η^2^ = 0.08). A total of 19 participants were above the clinical cut-off, 15 who had repeatedly covered COVID-19 stories (30%) and 4 who had not (9.8%). A further 17 participants met the criteria for sub-threshold PTSD, 11 (22%) who had repeatedly covered COVID-19 stories and 6 who had not (15%). This is according to the five symptoms definition of subthreshold PTSD that requires the participant to endorse at least one symptom for each criterion scoring two or above for the endorsed symptom [14].

Depression Symptoms. There were no significant differences in scores for depression symptoms between the two groups (F(180) = 2.47, *p* = 0.120). This did not change when trauma exposure and gender were added as covariates.

Psychological Distress. The groups differed significantly with respect to psychological distress (F(1116) = 5.20, *p* = 0.024), with those reporting to repeatedly cover COVID-19 stories scoring significantly higher than those who did not (small effect, η^2^ = 0.04). The effect was non-significant when trauma exposure and gender were added as a covariates (F(3113) = 1.57, *p* = 0.201).

Responses to Unwanted Memories. Journalists repeatedly reporting on COVID-19 were significantly more likely to ruminate in response to unwanted memories (F(189) = 7.87, *p* = 0.006). There were no differences between the groups in the use of numbing (F(189) = 3.63, *p* = 0.060) or suppression strategies in response to unwanted memories (F(189) = 1.92, *p* = 0.169). This did not change when trauma exposure and gender were added as covariates.

### 3.3. Predictors

Multiple linear regression was conducted on the whole sample to determine whether coping strategies (rumination, numbing and suppression) predicted PTSD symptoms and whether they interacted with trauma exposure as a predictor. Results of the regressions suggest there were no significant interaction effects (all *p* > 0.30). The subscales were then added as predictors whilst controlling for trauma exposure and gender. The final model was significant (F(582) = 18.31, *p* < 0.001) with a large effect, R^2^ = 0.53. See Table 2.

### 3.4. Protective Factors

Since intrusive re-experiencing symptoms are the hallmark feature of PTSD and predict the development and maintenance of the disorder [15], we investigated this phenomenon more closely in our sample in relation to resilience to identify potential protective factors. We compared those who covered COVID-19 repeatedly without developing re-experiencing symptoms with those who similarly reported on distressing COVID-19 news and who did develop re-experiencing symptoms. Those who did not report re-experiencing symptoms demonstrated significantly higher resilience scores on the CD-RISC (F(140) = 4.92, *p*= 0.032). We then compared the two groups on resilience appraisals related to the capacity to adapt to change since such appraisals reflect perceived self-efficacy and could be targeted with training. There were significant differences for each of the appraisals: “I am able to adapt when changes occur” (F(140) = 9.46, *p* = 0.004), “I can deal with whatever comes my way” (F(140) = 9.77, *p* = 0.003), “Having to cope with stress can make me stronger” (F(140) = 5.41, *p* = 0.025) and “I tend to bounce back after illness, injury or other hardships” (F(140) = 4.58, *p* = *0*.039).

## 4. Discussion

Our study found that journalists repeatedly reporting on the COVID-19 pandemic and its associated consequences had higher levels of PTSD symptoms and psychological distress than journalists who were working during the pandemic yet covering stories other than COVID-19. This is consistent with recent research that demonstrates high psychological distress among journalists working for international news organisations regularly reporting on COVD-19 [16] and consistent with previous research that shows repeated trauma exposure is a predictor for PTSD in journalists [4,17,18,19]. The prolonged and ever-changing nature of the pandemic has resulted in journalists being forced to deal with stories related to human suffering over and over again. The high level of PTSD symptoms, although not above the clinical cut off, appears to have an impact on journalists in this study, as evidenced by greater psychological distress for journalists reporting on COVID-19 than those who covered other news. Although PTSD cannot be diagnosed with self-report measures, the rate of a probable diagnosis in the group covering COVID-19 (30%) is equivalent to that found in Feinstein et al.’s study on war correspondents (28.6%) [1]. The group repeatedly covering COVID-19 stories also had higher levels of subthreshold PTSD, which is known to be long-lasting and can result in significantly greater impairment than those without symptoms [20]. The rate of probable PTSD in the whole sample was lower, at 6.3%, but still higher than that of the UK average (4%) and similar to that found in domestic American news journalists (7%) [21,22]. 

Whilst we cannot rule out whether responses to unwanted memories, such as rumination and numbing, maintain or predict PTSD severity in this sample, the findings suggest such strategies may be a useful target in treatment or a potential focus in the development of preventative interventions. Previous research has shown that it is possible to modify rumination through techniques, such as concreteness training [23]. Research on modifying risk factors is being conducted in other at-risk groups and is currently lacking in journalists [24]. Interventions are especially important for the journalism sector because of a perceived workplace culture that disincentivises disclosure of mental health problems [25]. 

The study also identifies resilience appraisals that appear to be protective for journalists, distinguishing those who reported on distressing COVID-19 news and who developed re-experiencing symptoms from those who reported on COVID-19 and did not develop re-experiencing symptoms. These appraisals related to the perceived capacity to adapt to change, can be modified with training, and are associated with improvement in PTSD symptoms, i.e., [12,24], adding to the body of research, which demonstrates it is possible to modify resilience with targeted interventions and thusly improve wellbeing see [26] for review. 

Our study has several limitations. We measured PTSD symptomatology rather than administering structured interviews to assess a PTSD diagnosis. Nonetheless, the level of symptomatology reported by journalists repeatedly covering COVID-19 stories equates to significant distress, as evidenced by high scores on our measure of psychological distress and the high rates of probable and subthreshold PTSD. The volume of COVID-19 coverage was measured in a dichotomous manner to make it easier for journalists to respond since previous research has demonstrated that journalists often struggle to estimate their workload and are more accurate at identifying high vs. low workloads [22]. It is also important to acknowledge the self-selected nature of the sample as we recruited via social media and newsletters. Future studies with larger sample sizes are needed.

## 5. Conclusions

Our study is the first to investigate potential factors that could protect journalists from developing PTSD when repeatedly reporting on news of human suffering. We found rates of probable PTSD to be high in this population and more likely for journalists who dwelled on or engaged in numbing strategies in response to unwanted trauma-related memories. Helpful resilience appraisals appeared to protect against re-experiencing symptoms following coverage of traumatic stories and may be a useful target for future interventions aimed at protecting the mental health of journalists. 

## Figures and Tables

**Table 1:** Demographic characteristics of participants and symptom measure scores.

	Repeatedly Covering COVID-19 Stories (*n* = 61)		Not Covering COVID-19 Stories (*n* = 59)		Total (*n* = 120)		*F*/*X*^2^	*p*
Age								
Mean (SD)	39.44	(10.74)	42.49	(11.72)	40.94	(11.29)	*F*(1119) = 2.21	0.140
Gender							*X*^2^(1, *n* = 120) = 5.65	0.017
Male	19	31.1%	3	52.5%	50	41.7%		
Female	42	68.9%	28	47.5%	70	58.3%		
Marital Status							*X*^2^(6, *n* = 120) = 10.92	0.091
Single	15	24.6%	8	13.6%	23	19.2%		
Married	23	37.7%	31	52.5%	54	45%		
Divorced/Separated	7	11.5%	1	1.7%	8	6.7%		
Widowed	1	1.6%	0	0%	1	0.8%		
Civil partnership	1	1.6%	1	1.7%	2	1.7%		
Long-term partner	14	23%	16	27.1%	30	25%		
Prefer not to say	0	0%	2	3.4%	2	1.7%		
Ethnicity							*X*^2^(1, *n* = 120) = 0.91	0.339
White British/European	47	77%	52	88.1%	99	82.5%		
Black/Indian/Asian/Arab	14	23%	7	11.9%	21	17.5%		
Mental Health								
Received diagnosis	24	39.2%	17	28.8%	41	34.2%	*X*^2^(1, *n* = 120) = 1.48	0.224
Received treatment	23	37.7%	17	28.8%	40	33.3%	*X*^2^(1, *n* = 120) = 0.73	0.394
Type of Employment							*X*^2^(1, *n* = 120) = 5.58	0.018
Freelance	11	18%	22	37.3%	33	27.5%		
Organisation	50	82%	37	62.7%	87	72.5%		
Job Title							*X*^2^(7, *n* = 120) = 7.43	0.386
Broadcast journalist	14	23%	9	15.3%	23	19.2%		
Video journalist	1	1.6%	4	6.8%	5	4.2%		
Reporter	26	42.6%	21	35.6%	47	39.2%		
Editor	12	19.7%	13	22%	25	20.8%		
Producer	4	6.6%	2	3.4%	6	5%		
Camera Operator	0	0%	1	1.7%	1	0.8%		
Other	4	6.6%	9	15.6%	13	10.8%		
Years in role							*F*(1,119) = 0.50	0.483
Mean (SD)	7.69	(6.81)	8.66	(8.15)	8.17	(7.48)		
Trauma Exposure (no. of lifetime incidents)							*F*(1,119) = 5.94	0.016
Mean (SD)	11.57	(5.62)	8.89	(6.12)	10.30	(5.98)		
COVID-19 Impact								
Had COVID-19	5	8.2%	2	3.4%	7	5.8%	*X*^2^(1, *n* = 120) = 1.26	0.261
Family/friend had COVID-19	20	32.8%	22	37.3%	42	35%	*X*^2^(1, *n* = 120) = 0.27	0.605
Financial difficulties due to COVID-19	12	19.7%	13	22%	25	20.8%	*X*^2^(1, *n* = 120) = 0.10	0.750
Family difficulties due to COVID-19	19	31.1%	15	25.4%	34	28.3%	*X*^2^(1, *n* = 120) = 0.48	0.487
Working longer hours	35	57.4%	23	39%	58	48.3%	*X*^2^(1, *n* = 120) = 4.06	0.044
Interviewed people with COVID-19	24	39.3%	8	13.6%	32	26.7%	*X*^2^(1, *n* = 120) = 10.20	0.001
Seen people suffer with COVID-19	18	29.5%	6	10.2%	24	20%	*X*^2^(1, *n* = 120) = 7.01	0.008
Symptom Measures Mean (SD)								
PCL-5	21.34	(18.20)	12.26	(12.41)	-	-	*F*(1,90) = 7.50	0.007
PHQ-9	7.86	(5.54)	6.03	(4.99)	-	-	*F*(1,80) = 2.47	0.120
GHQ-12	29.70	(5.91)	27.09	(6.54)	-	-	*F*(1,116) = 5.20	0.024
Process Measures Mean (SD)								
RIQ Total Score	21.65	(10.40)	16.14	(8.87)	-	-	*F*(1,89) = 7.26	0.008
Rumination	8.37	(5.97)	5.19	(4.61)	-	-	*F*(1,89) = 7.87	0.006
Suppression	9.29	(4.20)	8.12	(3.76)	-	-	*F*(1,89) = 1.92	0.169
Numbing	4.00	(3.12)	2.83	(2.55)	-	-	*F*(1,89) = 3.63	0.060

PCL-5: PTSD Checklist 5. PHQ-9: Patient Health Questionnaire-9. GHQ-12: General Health Questionnaire-12. RIQ: Responses to Intrusions Questionnaire.

**Table 2:** Confidence intervals and standard errors based on 1000 bootstrap samples.

	*b*	*SE B*	*ß*	*p*
Step 1				
Gender	5.76	3.66	0.17	0.120
Total number of trauma exposures	0.37	0.31	0.13	0.235
Step 2				
Gender	3.69	2.68	0.11	0.173
Total number of trauma exposures	0.37	0.23	0.13	0.105
Rumination	1.49	0.24	0.52	0.001
Thought Suppression	−0.49	0.40	−0.12	0.223
Numbing	1.94	0.56	0.36	0.001

Note. *R*^2^ = 0.057 for step 1; Δ*R*^2^ for step 2.

## Data Availability

The data presented in this study are available on request from the corresponding author. The data are not publicly available due to privacy reasons.
