# Correction: Tyson, G.; Wild, J. Post-Traumatic Stress Disorder Symptoms among Journalists Repeatedly Covering COVID-19 News. *Int. J. Environ. Res. Public Health* 2021, *18*, 8536

**DOI:** 10.3390/ijerph182111235

**Published:** 2021-10-26

**Authors:** Gabriella Tyson, Jennifer Wild

**Affiliations:** Oxford Centre for Anxiety Disorders and Trauma, Department of Experimental Psychology, University of Oxford, Oxford OX1 1TW, UK; jennifer.wild@psy.ox.ac.uk

## Error in Figure/Table

In the original article [1], there was an error in Table 1 as published. The General Health Questionnaire (GHQ) was scored with a 1–4, rather than a 0–3, scale. The altered scores do not affect the subsequent analysis or results. The corrected Table 1 appears below. The authors apologize for any inconvenience caused and state that the scientific conclusions are unaffected. The original article has been updated.

## Figures and Tables

**Table 1:** Demographic characteristics of participants and symptom measure scores.

	Repeatedly Covering COVID-19 Stories (*n* = 61)		Not Covering COVID-19 Stories (*n* = 59)		Total (*n* = 120)		*F*/*X*^2^	*p*
Age								
Mean (SD)	39.44	(10.74)	42.49	(11.72)	40.94	(11.29)	*F*(1,119) = 2.21	0.140
Gender							*X*^2^(1, *n* = 120) = 5.65	0.017
Male	19	31.1%	31	52.5%	50	41.7%		
Female	42	68.9%	28	47.5%	70	58.3%		
Marital Status							*X*^2^(6, *n* = 120) = 10.92	0.091
Single	15	24.6%	8	13.6%	23	19.2%		
Married	23	37.7%	31	52.5%	54	45%		
Divorced/Separated	7	11.5%	1	1.7%	8	6.7%		
Widowed	1	1.6%	0	0%	1	0.8%		
Civil partnership	1	1.6%	1	1.7%	2	1.7%		
Long-term partner	14	23%	16	27.1%	30	25%		
Prefer not to say	0	0%	2	3.4%	2	1.7%		
Ethnicity							*X*^2^(1, *n* = 120) = 0.91	0.339
White British/European	47	77%	52	88.1%	99	82.5%		
Black/Indian/Asian/Arab	14	23%	7	11.9%	21	17.5%		
Mental Health								
Received diagnosis	24	39.2%	17	28.8%	41	34.2%	*X*^2^(1, *n* = 120) = 1.48	0.224
Received treatment	23	37.7%	17	28.8%	40	33.3%	*X*^2^(1, *n* = 120) = 0.73	0.394
Type of Employment							*X*^2^(1, *n* = 120) = 5.58	0.018
Freelance	11	18%	22	37.3%	33	27.5%		
Organisation	50	82%	37	62.7%	87	72.5%		
Job Title							*X*^2^(7, *n* = 120) = 7.43	0.386
Broadcast journalist	14	23%	9	15.3%	23	19.2%		
Video journalist	1	1.6%	4	6.8%	5	4.2%		
Reporter	26	42.6%	21	35.6%	47	39.2%		
Editor	12	19.7%	13	22%	25	20.8%		
Producer	4	6.6%	2	3.4%	6	5%		
Camera operator	0	0%	1	1.7%	1	0.8%		
Other	4	6.6%	9	15.6%	13	10.8%		
Years in role							*F*(1,119) = 0.50	0.483
Mean (SD)	7.69	(6.81)	8.66	(8.15)	8.17	(7.48)		
Trauma Exposure (no. of lifetime incidents)							*F*(1,119) = 5.94	0.016
Mean (SD)	11.57	(5.62)	8.89	(6.12)	10.30	(5.98)		
COVID-19 Impact								
Had COVID-19	5	8.2%	2	3.4%	7	5.8%	*X*^2^(1, *n* = 120) = 1.26	0.261
Family/Friend had COVID-19	20	32.8%	22	37.3%	42	35%	*X*^2^(1, *n* = 120) = 0.27	0.605
Financial difficulties due to COVID-19	12	19.7%	13	22%	25	20.8%	*X*^2^(1, *n* = 120) = 0.10	0.750
Family difficulties due to COVID-19	19	31.1%	15	25.4%	34	28.3%	*X*^2^(1, *n* = 120) = 0.48	0.487
Working longer hours	35	57.4%	23	39%	58	48.3%	*X*^2^(1, *n* = 120) = 4.06	0.044
Interviewed people with COVID-19	24	39.3%	8	13.6%	32	26.7%	*X*^2^(1, *n* = 120) = 10.20	0.001
Seen people suffer with COVID-19	18	29.5%	6	10.2%	24	20%	*X*^2^(1, *n* = 120) = 7.01	0.008
Symptom Measures Mean (SD)								
PCL-5	21.34	(18.20)	12.26	(12.41)	-	-	*F*(1,90) = 7.50	0.007
PHQ-9	7.86	(5.54)	6.03	(4.99)	-	-	*F*(1,80) = 2.47	0.120
GHQ-12	17.70	(5.91)	15.09	(6.54)	-	-	*F*(1,116) = 5.20	0.024
Process Measures Mean (SD)								
RIQ total score	21.65	(10.40)	16.14	(8.87)	-	-	*F*(1,89) = 7.26	0.008
Rumination	8.37	(5.97)	5.19	(4.61)	-	-	*F*(1,89) = 7.87	0.006
Suppression	9.29	(4.20)	8.12	(3.76)	-	-	*F*(1,89) = 1.92	0.169
Numbing	4.00	(3.12)	2.83	(2.55)	-	-	*F*(1,89) = 3.63	0.060

PCL-5: PTSD Checklist 5. PHQ-9: Patient Health Questionnaire-9. GHQ-12: General Health Questionnaire-12. RIQ: Responses to Intrusions Questionnaire.
